# Human herpesvirus 6A induces apoptosis of primary human fetal astrocytes via both caspase-dependent and -independent pathways

**DOI:** 10.1186/1743-422X-8-530

**Published:** 2011-12-12

**Authors:** Bin Gu, Guo-Feng Zhang, Ling-Yun Li, Feng Zhou, Dong-Ju Feng, Chuan-Lin Ding, Jing Chi, Chun Zhang, Dan-Dan Guo, Jing-Feng Wang, Hong Zhou, Kun Yao, Wei-Xing Hu

**Affiliations:** 1 Department of Neurosurgery, First Affiliated Hospital of Nanjing Medical University, Nanjing 210029, China; 2 Department of Microbiology and Immunology, Nanjing Medical University, Nanjing 210029, China; 3 Tumor Immunobiology Program, James Graham Brown Cancer Center, University of Louisville, Louisville, KY 40202, USA

**Keywords:** Apoptosis, Human herpesvirus 6A, Primary human fetal astrocyte, Caspase

## Abstract

**Background:**

Human herpesvirus 6 (HHV-6) is a T-lymphtropic and neurotropic virus that can infect various types of cells. Sequential studies reported that apoptosis of glia and neurons induced by HHV-6 might act a potential trigger for some central nervous system (CNS) diseases. HHV-6 is involved in the pathogenesis of encephalitis, multiple sclerosis (MS) and fatigue syndrome. However, the mechanisms responsible for the apoptosis of infected CNS cells induced by HHV-6 are poorly understood. In this study, we investigated the cell death processes of primary human fetal astrocytes (PHFAs) during productive HHV-6A infection and the underlying mechanisms.

**Results:**

HHV-6A can cause productive infection in primary human fetal astrocytes. Annexin V-PI staining and electron microscopic analysis indicated that HHV-6A was an inducer of apoptosis. The cell death was associated with activation of caspase-3 and cleavage of poly (ADP-ribose) polymerase (PARP), which is known to be an important substrate for activated caspase-3. Caspase-8 and -9 were also significantly activated in HHV-6A-infected cells. Moreover, HHV-6A infection led to Bax up-regulation and Bcl-2 down-regulation. HHV-6A infection increased the release of Smac/Diablo, AIF and cytochrome c from mitochondria to cytosol, which induced apoptosis via the caspase-dependent and -independent pathways. In addition, we also found that anti-apoptotic factors such as IAPs and NF-κB decreased in HHV-6A infected PHFAs.

**Conclusion:**

This is the first demonstration of caspase-dependent and -independent apoptosis in HHV-6A-infected glial cells. These findings would be helpful in understanding the mechanisms of CNS diseases caused by HHV-6.

## Background

Human herpesvirus 6 (HHV-6), a member of the beta herpesvirus family, is a T-lymphotropic virus and the causal agent of exanthema subitum [1-3]. In recent studies, HHV-6 has been detected in numerous central nervous system (CNS) diseases including encephalitis, multiple sclerosis, temporal lobe epilepsy and glioma [4-7]. These findings suggest that HHV-6 may be associated with some CNS diseases. In vitro, HHV-6 has been shown to infect human glial cells (microglia, oligodendrocytes and astrocytes) and induce apoptosis [8-10]. However, the molecular mechanisms of apoptosis induced by HHV-6 in glial cells are not fully understood as yet.

Apoptosis, a programmed suicide death of cells, which is characterized by chromatin condensation, DNA fragmentation, membrane blebbing, and cell shrinkage, can occur through the intrinsic and extrinsic casepase pathways [11]. Caspases, a family of cysteine proteases, regulate the initiation and the final execution of apoptosis in receptor-mediated and mitochondria-mediated pathways [12]. In the receptor-mediated pathway, caspase-8 is the initiator caspase that can directly activate the final executioner caspase-3 [13]. In the mitochondria-mediated pathway, mitochondria release several pro-apoptotic factors including cytochrome c, Smac/Diablo, and apoptosis-inducing factor (AIF) into the cytosol [14]. Cytosolic cytochrome c binds with apoptotic protease activating factor 1 (APAF1) to produce active caspase-9 and subsequently active caspase-3 for caspase-dependent apoptosis. Samc/Diablo is an antagonistic protein for inhibitor of apoptosis proteins (IAPs), promotes apoptosis along with cytochrome c by activating caspases [15]. Mitochondria-mediated apoptosis may also occur in caspase-independently way after mitochondrial release of AIF that is translocated to the nucleus for induction of chromatin condensation and DNA fragmentation [16].

In the present study, we investigated the effect and molecular mechanism of HHV-6A inducing apoptosis in primary human fetal astrocytes (PHFAs). We found that HHV-6A induced apoptosis in PHFAs through both caspase-dependent and -independent apoptotic pathways. In addition, our finding also demonstrated that HHV-6A could promote cell death by suppressing IAPs and NF-κB-mediated anti-apoptosis pathways. To our knowledge, this is the first demonstration of the mechanisms of apoptosis induced by HHV-6A in astrocytes.

## Results

### HHV-6A causes productive infection in PHFAs

**Figure 1:**
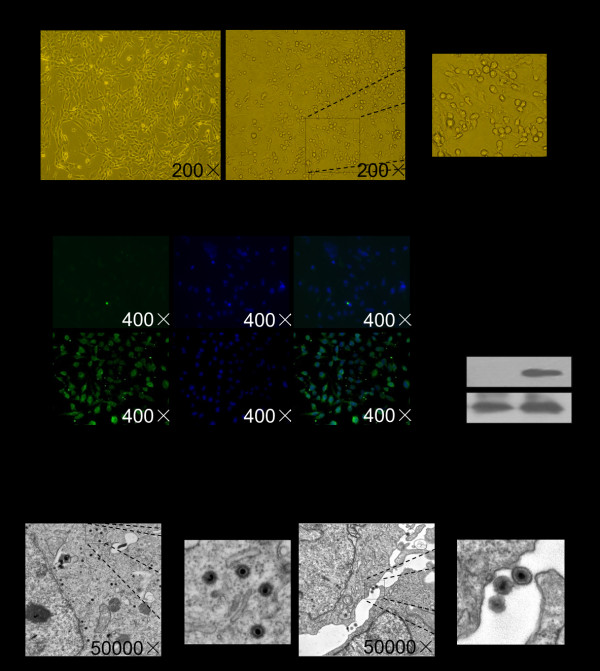
**HHV-6A causes infection in PHFAs**. **a**. HHV-6A infection exhibited typical cytopathic effects in infected PHFAs. The morphological characteristics of PHFAs infected with or without HHV-6A were observed under light microscope. **b**. HHV-6A-infected PHFAs express viral gp60/110 protein at 72 h post-infeciton. The gp60/110 protein was determined by IFA and western blotting with an anti-gp60/110 monoclonal antibody. **c**. Electron microscopic photographs of typical herpesvirus-like particles were observed in both cytoplasm and extracellular matrix of HHV-6A-infected PHFAs.

HHV-6A was used to infect PHFAs at comparable levels of virus DNA (1 × 10^8 ^copies/10^6 ^cells) as determined by quantitative PCR. HHV-6A-infected PHFAs showed typical cytopathic effects (CPE) such as cellular swelling and cell fusion at 72 h post-infection (hpi) (Figure 1a). To further determine HHV-6A infection in PHFAs, the expression of a late protein gp60/110 was analyzed using immunofluorescence assay and western blotting at 72 hpi. As shown in Figure 1b, a prominent expression of HHV-6 gp60/110 was detected in HHV-6A-infected PHFAs compared with that in the control mock-infected cells. The gp60/110 late protein was clearly localized in the cytoplasm of most multinucleate giant cells. Electron microscopic analyses were also performed on HHV-6A-infected PHFAs at 72 hpi. As shown in Figure 1c, viral particles could be visualized in both cytoplasm and extracellular matrix of HHV-6A-infected PHFAs. These results indicate that HHV-6A can cause productive infection in PHFAs.

### HHV-6A induces apoptosis of PHFAs

**Figure 2:**
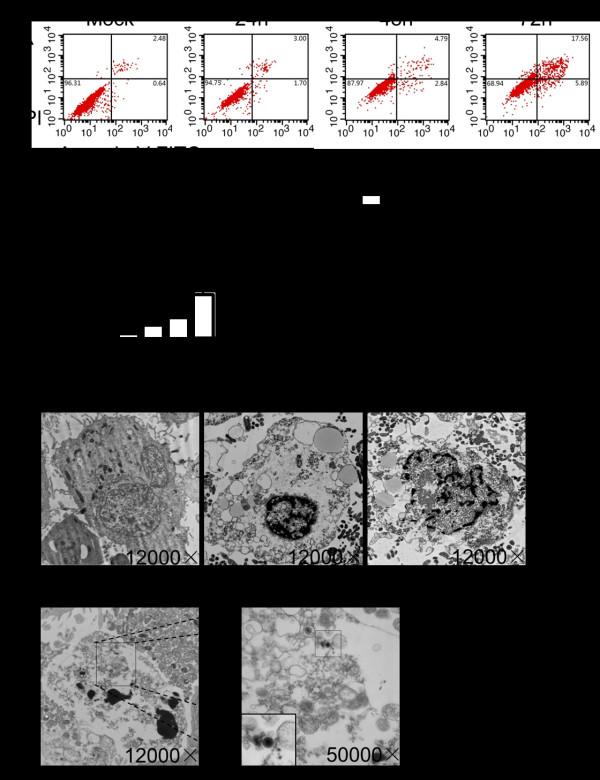
**HHV-6A infection induces apoptosis of PHFAs**. **a**. Mock- and HHV-6A-infected PHFAs were stained with annexin V-PI and analyzed by flow cytometry. Percentage of apoptotic cells was summarized. Each column represents the mean ± SD of three independent experiments (*P < 0.05, **P < 0.01, ***P < 0.001). **b**. Electron microscopic photographs of mock- and HHV-6A-infected PHFAs. **c**. Electron microscopic photographs of virus-like particles in apoptotic HHV-6A-infected PHFAs.

To investigate the effect of HHV-6A infection on apoptosis in PHFAs, cells infected with HHV-6A were stained with annexin-V-FITC and propidium iodide (PI) after 24, 48, and 72 hpi and analyzed by flow cytometry. As shown in Figure 2a, we observed a high percentage of annexin-positive cells (apoptotic cells) in HHV-6A-infected cells at 72 hpi compared to mock-infected cells. The percentage of early apoptotic cells and late apoptotic cells at 72 hpi reached 5.89% and 17.5% compared to 0.64% and 2.48% in mock-infected cells, respectively. To further confirm the effect of HHV-6A on cell apoptosis, we also observed the morphologic changes in HHV-6A-infected cells using transmission electron microscopy. HHV-6A-infected PHFAs showed the typical features of cell apoptosis: marginalized and condensed chromatin matrix, shrinkage and blebbing of the cytoplasm and fragmented nuclei (Figure 2b). Virus-like particles could be found in apoptotic HHV-6A-infected PHFAs (Figure 2c).

### HHV-6A triggers caspases activation

**Figure 3:**
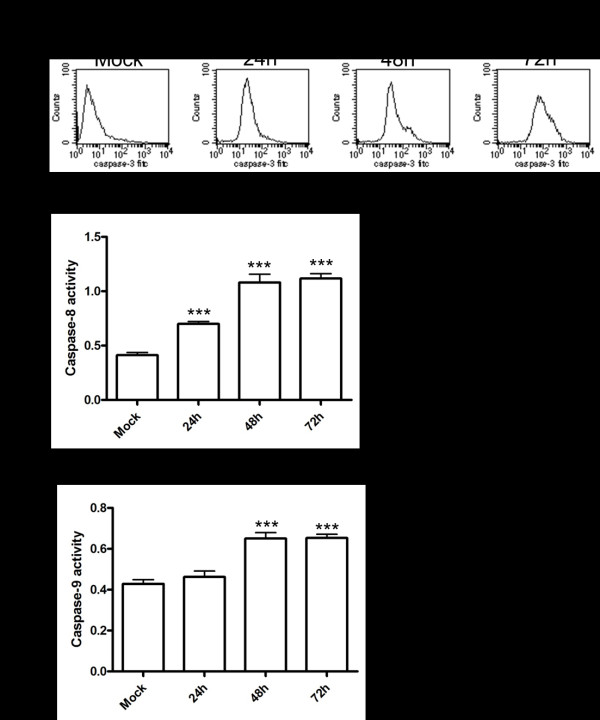
**HHV-6A triggers caspases activation**. **a**. Mock- and HHV-6A-infected PHFAs were collected at various time points and the levels of activated caspase-3 were measured by flow cytometry. **b-c**. The activation of caspase-8 and caspase-9 was examined by colorimetric method using lysates from mock-infected and HHV-6A-infected PHFAs. Each column represents the mean ± SD of three independent experiments (***P < 0.001).

Caspases are synthesized as inactive precursors that are processed to large and small subunits to form the active enzymes. Caspase-3 is one of the main effective caspases, which are activated in response to both intracellular and extracellular death signals. To explore the pathway by which HHV-6A induced apoptosis, we measured caspase-3 activity in HHV-6A-infected PHFAs with anti-active caspase-3 antibody using flow cytometry. PHFAs with activated caspase-3 were about 2.81%, 10.12% and 19.31% at 24, 48 and 72 hpi, respectively, whereas the value was only 0.69% in the mock-infected cells (Figure 3a). To further define whether HHV-6A induces apoptosis via the receptor-mediated or the mitochondria-mediated pathways, the activities of caspase-8 and -9 were measured, respectively. As shown in Figure 3b, c, HHV-6A infection resulted in significant increases in caspase-8 and caspase-9 activities at 48 and 72 hpi in HHV-6A-infected cells compared with mock-infected cells. These data indicate that HHV-6A induce apoptosis of PHFAs by both the receptor-mediated and the mitochondria-mediated pathways.

### HHV-6A activates PARP cleavage and up-regulates bax/bcl-2 ratio

**Figure 4:**
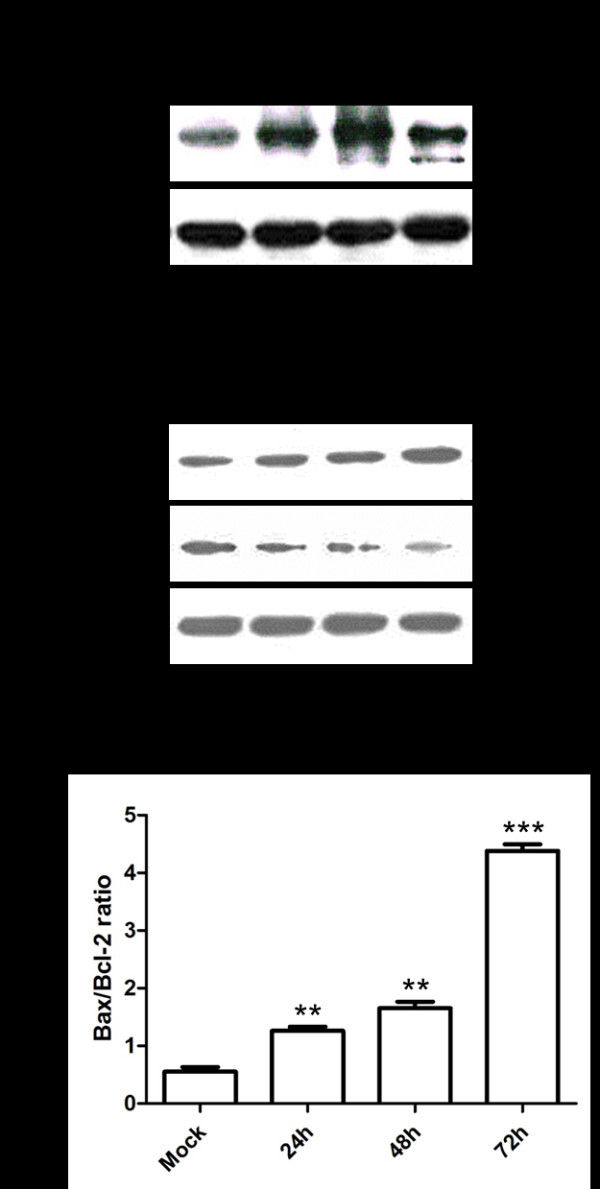
**HHV-6A activates PARP cleavage and up-regulates Bax/Bcl-2 ratio**. **a**. PARP in mock-infected and HHV-6A-infected cells was analyzed by Western blotting. **b**. Expressions of Bcl-2 and Bax were detected by Western blots using anti-Bcl-2 and anti-Bax antibodies, respectively. β-actin was used as a loading control. Quantitative values of Bcl-2 and Bax are the mean ± SD from three independent experiments (**P < 0.01, ***P < 0.001).

PARP is an established substrate for caspase-3 in the apoptotic events. Cleavage of PARP facilitates cellular disassembly and serves as a marker of cells undergoing apoptosis. Western blotting was used to detect endogenous full-length PARP (116 KD), as well as the large fragment (89 KD) of PARP resulting from caspase cleavage. As shown in Figure 4a, the 89 KD cleaved fragment of PARP was detected in infected cells at 48 and 72 hpi, but not detected in the mock-infected cells.

The mitochondria-mediated pathway of apoptosis is regulated by the Bcl-2 family proteins, which are known to directly regulate mitochondrial membrane permeability. We examined the levels of expression of Bax (pro-apoptotic) and Bcl-2 (anti-apoptotic) proteins using Western blotting analysis. As shown in Figure 4b, the levels of Bcl-2 protein were significantly decreased following HHV-6A infection compared to that in mock-infected cells, whereas the expression of Bax protein was significantly increased in HHV-6A-infected cells. These results indicate Bax/Bcl-2 ratio was significantly increased in HHV-6A-infected cells compared with mock-infected cells.

### HHV-6A infection results in the release of pro-apoptotic proteins from mitochondria

**Figure 5:**
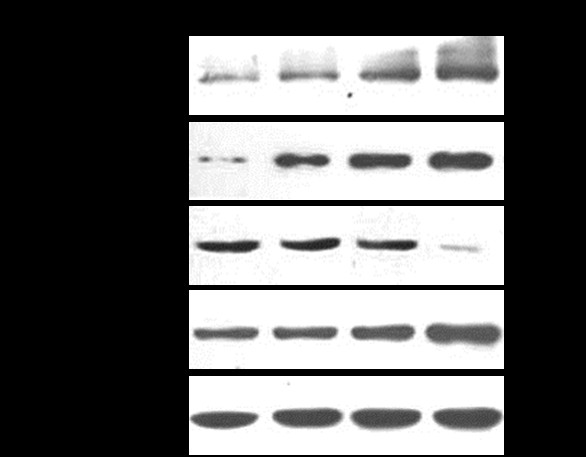
**HHV-6A infection results in the release of pro-apoptotic proteins from mitochondria**. Expressions of pro-apoptotic proteins liberated from mitochondria were detected by Western blots as described in Methods and Materials. β-actin was used as a loading control.

Mitochondria may release several molecules including cytochrome c, Smac/Diablo, and AIF to induce apoptosis. Mitochondrial cytochrome c release is a well-known pre-condition for formation of apoptosome and activation of caspases for apoptosis. As shown in Figure 5, there is a marked increase in the levels of cytochrome c released from mitochondria to cytoplasm at 48 and 72 hpi compared with control. Smac/Diablo plays an important role in apoptosis by down-regulation anti-apoptotic IAPs. The expression levels of Smac/Diablo were significantly increased following HHV-6A infection in a time-dependent manner compared to that in mock-infected cells. In addition, the expression levels of AIF, determining caspase-independent pathway of apoptosis were also increased obviously in HHV-6A infected cells compared to that in mock-infected cells. The results suggest that HHV-6A infection in PHFAs can provoke apoptosis via the mitochondrial intrinsic pathway.

### HHV-6A suppresses IAPs and NF-κB-mediated anti-apoptosis effect

**Figure 6:**
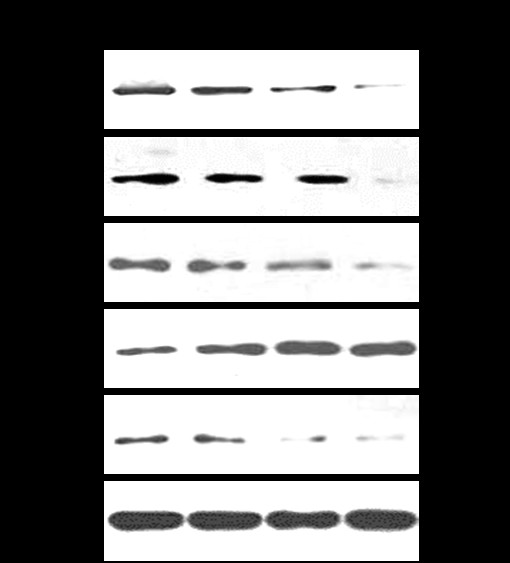
**Down-regulation of anti-apoptotic proteins by HHV-6A infection**. Representative Western blots show levels of expression of NF-κB, IκBα, c-IAP1, c-IAP2 and XIAP. β-actin was used as a loading control.

IAPs are thought to function primarily by negative regulation caspases, which are cysteine proteases involved in apoptosis. In human cells, IAPs mainly include cIAP1, cIAP2 and XIAP. As shown in Figure 6, the levels of these three IAPs were significantly decreased in the HHV-6A-infected cells compared to those in mock-infected cells.

NF-κB plays a crucial role not only in immunity, inflammation and cell migration but also in cell survival and apoptosis. Many studies confirmed that NF-κB up-regulation and activation exerted an anti-apoptotic effect leading to cells survival, transformation, and resistance to radiation and chemotherapy [17]. As shown in Figure 6, the levels of NF-κB were significantly decreased in HHV-6A-infected cells compared to that in mock-infected cells, while the expression of NF-κB inhibitor-IκBα protein was significantly increased in HHV-6A-infected cells. These results indicate that HHV-6A infection injures IAPs and NF-κB-mediated anti-apoptosis signal pathways in PHFAs.

## Discussion

HHV-6 was first isolated from peripheral blood mononuclear cells of patients with lymphoproliferative disorders and AIDS [2]. There are two variants of HHV-6 (A and B) according to distinct genetic, immunological and virological characteristics [18]. As with other virus, HHV-6 is able to induce apoptosis of host cells. Subsequent studies have demonstrated that HHV-6 has been shown to induce apoptosis in astrocytes, oligodendrocytes, neuronal cell lines and CD4^+ ^T lymphocytes [8,19,20]. Gardell et al. [8] reported that HHV-6A induced apoptosis by an unknown mechanism in astrocytes, oligodendrocytes and neuronal cell lines. Inoue et al. [20] demonstrated that TNF-α and anti-Fas antibodies augmented HHV-6-induced apoptosis, suggesting an involvement bof death receptors in HHV-6-induced apoptosis. In contrast, Inchimi et al. [21] found that HHV-6 induced apoptosis of cord blood lymphocytes through a receptor-independent pathway.

Caspases play a critical role in apoptosis, which cleave specific substrates and activate downstream molecules and culminate in cell death [11,13]. However, the roles of caspases in HHV-6-induced apoptosis of astrocytes haven't been studied yet. In this study, we demonstrated that the activities of caspase-3, -8 and -9 were all increased in HHV-6A-induced apoptosis of PHFAs. In addition, we found that PARP was cleaved in HHV-6A-induced apoptotic PHFAs. Caspase-3 is a common effector of both death receptor and the mitochondrial signaling pathways. Caspase-8 is activated by the death receptor signaling pathway, whereas caspase-9 is activated in the mitochondrial signaling pathway during apoptosis. We speculate that HHV-6A-induced apoptosis in astrocytes via both caspase-dependent receptor and mitochondrial apoptotic pathways.

Bcl-2 family proteins are central regulators of the mitochondrial apoptotic pathway and have been implicated in various models of virus-induced apoptosis. Pugazhenthi et al. [22] found that simian varicella virus induced apoptosis in monkey kidney cells via caspase-dependent mitochondrial pathway and involves down-regulation of bcl-2 expression. The translocation and accumulation of Bax, a pro-apoptotic factor of Bcl-2 family in mitochondria will lead to release of cytochrome c and AIF [23]. We examined the expression of Bcl-2 and Bax in HHV-6A-induced mitochondrial dysfunction. Our data showed that the anti-apoptotic protein Bcl-2 decreased, which was accompanied by the increase of pro-apoptotic protein Bax during HHV-6A infection, suggesting that Bcl-2 and Bax were involved in the apoptosis of HHV-6A-infected PHFAs.

Up-regulation of Bax induces the permeabilization of mitochondrial outer membrane and initiates mitochondrial dysfunction. Mitochondria may release several molecules including cytochrome c, Smac/Diablo, and AIF to induce apoptosis via the caspase-dependent and -independent pathways [24-26]. We separated the cytosolic and mitochondrial fractions to examine the cytochrome c levels by Western blotting and found a marked increase in cytochrome c level in the cytosolic fraction due to a concomitant decrease in the cytochrome c level in the mitochondrial fraction following HHV-6A infection. Mitochondrial cytochrome c releases into cytoplasm to bind to the apoptosis protease activation factor (APAF1) and to form a complex of apoptosome activating pro-caspase 9. The activation of pro-caspase 9 initiates an enzymatic reaction cascade leading to the execution of apoptosis in cells [27]. We also observed a time-dependent increase the cytosolic level of Smac/Diablo following infection compared with control cells. Smac/Diablo can inhibit inhibitor-of-apoptosis-proteins (IAPs), which otherwise inactivate caspases [28]. Mitochondria-mediated apoptosis may also occur caspase-independently after mitochondrial release of AIF and Endo G which are translocated to the nucleus for induction of chromatin condensation and DNA fragmentation. Our investigation demonstrated that HHV-6A markedly increased the cytosolic level of AIF in PHFAs, indicating involvement of caspase-independent pathway of apoptosis [29].

In addition, NF-κB reportedly induces the expression of c-IAP1, c-IAP2 and XIAP, thereby promoting NF-κB activation in a positive feed-back system [30]. NF-κB up-regulation exerts an anti-apoptotic effect leading to cells survival, transformation, and resistance to radiation and drug therapies [31]. In contrast, NF-κB down-regulation will break this feed-back loop and reduce the expression of c-IAP1, c-IAP2 and XIAP, which are the direct caspase inhibitors. In the present study, we found that HHV-6A decreased NF-κB and increased Iκ-Bα expression in time-dependent manners. IκBα is one member of the family of cellular proteins that function to inhibit the activity of NF-κB. IκBα inhibits NF-κB by masking the nuclear localization signals of NF-κB proteins and keeping them sequestered in an inactive state in the cytoplasm. IκBα up-regulation may inhibit the activity of NF-κB which was observed down-regulation in HHV-6A-infected PHFAs. We also found that HHV-6A decreased expression of c-IAP1, c-IAP2 and XIAP. Increased mitochondrial release of Smac/Diablo could antagonize IAPs expression. Suppression of survival factors such as IAPs and NF-κB could be due to cytosolic up-regulation of Smac/Diablo [17].

## Conclusion

We demonstrated that HHV-6A induces cell apoptosis in PHFAs through both caspase-dependent and -independent apoptosis pathways, as evidenced by (1) activation of caspase-3, -8 and -9; (2) increasing the ratio of Bax/Bcl-2; (3) increasing the presence of Smac/Diablo, AIF and cytochrome c in cytoplasm; (4) down-regulation of anti-apoptotic NF-κB and IAPs. The identification of the apoptotic signaling pathways in HHV-6A-infected PHFAs would be very helpful in understanding the mechanisms by which HHV-6A infection causes diseases in the CNS.

## Materials and methods

### Cells and viruses

The primary human fetal astrocytes (Sciencell) were cultured in DEME/F12 (Hyclone) supplemented with 10% fetal calf serum, 100 IU/ml penicillin/streptomycin (Invitrogen). Human T-cell line HSB-2 cells were cultured in RPMI 1640 medium containing 8% fetal calf serum. HHV-6A strain GS was inoculated into HSB-2 cells. The cells were frozen and thawed twice when 80% of HHV-6A-infected HSB-2 cells showed the cytopathic effects (CPE), then centrifuged at 2000 × g for 10 min. The supernatants were stored at -70°C as cell-free virus. Viral DNA equivalents of the frozen aliquot were tested by quantitative PCR. Uninfected HSB-2 cells were similarly cultured and treated using the same procedure and used for mock infection. For infection, 3 × 10^5 ^primary human fetal astrocytes (PHFAs) were seeded onto a poly-L-lysine (Sigma)-coated 25-cm2 flask (Corning). After an overnight incubation, the plate was washed three times with phosphate-buffered saline (PBS) and infected with cell-free supernatant containing 10^8 ^viral DNA copies/10^6 ^PHFAs. After 3 h incubation at 37°C in 5% CO_2_, cultures were washed three times with PBS, and fresh medium was added. The HHV-6A-infected cells were checked for CPE every day in microscopy. Procedures for mock infection were performed in the same manner as for viral infection.

### Immunofluorescence assay (IFA)

PHFAs were cultured on poly-L-lysine-coated 2-chamber glass slides, and the infection was performed as described above. The procedure of immunofluorescence assay has previously been described [32]. Briefly, PHFAs infected with or without HHV-6A were fixed in 4% paraformaldehyde (in PBS), permeabilized in 0.5% Triton X-100 (in PBS), and stained with the anti-gp60/110 monoclonal antibodies (Chemicon international) followed by secondary antibody labeled with fluorescein isothiocyanate (FITC).

### Electron microscopy

Cells were fixed with 2.5% glutaradehyde at room temperature for 1 h. After washing with PBS, the cells were collected, dehydrated in a series of 70%, 80% and 90% ethanol, and embedded in Epon. Ultrathin sections were cut and mounted in nickel grids, stained with uranyl acetate and lead citrate, and examined by a transmission electron microscopy.

### Annexin V-propidium iodide (PI) staining

Apoptosis was measured by Annexin V-propidium iodide (PI) staining and flow cytometry. Infected and uninfected PHFAs were trypsinized, washed in PBS and incubated with Annexin V-FITC and PI solution (Bender MedSystems, Burlingame) in the dark for 15 min. Samples were analyzed by flow cytometry with FACSCalibur and BD CellQuest Pro software (Becton Dickinson, Mountain View). The amount of early apoptosis and late apoptosis was determined as the percentage of Annexin V^+^/PI^- ^and Annexin V^+^/PI^+^, respectively.

### Analysis of activated caspase-3 by flow cytometry

Caspase-3 activities in HHV-6A-infected and mock-infected PFHA were tested by flow cytometry with FITC-DEVD-FMK that recognizes cleaved caspase-3 according to the protocol by the manufacturer (Biovision Inc.).

### Analysis of caspase-8 and caspase-9 using a colorimetric method

The activation of caspase-8 and caspase-9 was analyzed using a colorimetric assay kit (KeyGEN). Briefly, mock-infected and HHV-6A-infected PFHA were collected and resuspended in 50 μl of lysis buffer and incubated on ice for 30 min. After centrifugation, the protein concentration was assayed by the BCA Protein Assay kit (Byotime), and 50 μg protein was diluted in 50 μl lysis buffer for each assay. Then 5 μl of casepase-8 or caspase-9 substrate were added, respectively. The reaction mixture was incubated at 37°C for 4 h. The released chromphore was measured at 405 nm by a microplate reader.

### Preparation of cytosolic and mitochondrial extracts

Cells were washed twice with PBS and kept for 1 h in ice-cold hypotonic buffer (20 mM HEPES, pH7.4, 1.5 mM MgCl2, 10 mM KCl, 1 mM EDTA, 1 mM EGTA, 1 mM dithiothreitol, 1 mM phenylmethylsulfonyl fluoride, 10 μg/ml leupeptin, aprotinin and pepstatin) containing 250 mM sucrose. The cells were homogenized using a Dounce homogenizer (KONTES), and cytosolic and mitochondrial extracts were isolated as described previously [33].

### Western blotting

Cells were lysed with a lysis buffer containing 50 mmol/L Tris (pH7.4), 0.5%NP-40, 0.01% SDS and a cocktail of protease inhibitors. Equal amounts of protein (30 μg) estimated by the BCA Protein Assay kit (Byotime) were separated by electrophoresis on 10% polyacrylamide gel and transferred to a PDVF membrane (Millipore). After blocking for 1 hour with 5% nonfat dry milk in TBST buffer containing 50 mmol/L Tris-HCl, 150 mmol/L NaCl and 0.1% Tween 20 (pH7.6) at room temperature, the blots were incubated overnight at 4°C with the specific primary antibody. Primary antibodies used were β-actin, IκBα, NF-κB, XIAP, c-IAP1, c-IAP2, PARP, AIF, Cytochrome c and Samc/Diablo (Cell Signaling Technology), Bax and Bcl-2 (Bioworld). Membranes were subsequently incubated with horseradish peroxidase-conjugated secondary antibody (Santa Cruz Biotechnology) for 1 h at room temperature and developed using a chemiluminescent (ECL) reagent (Applygen Technologies). The results were scanned using Gel Imaging System (UVP Company) and measured using Gel-Pro Analyzer software (Media Cybernetics).

### Statistical analysis

Data were presented as means ± SD. One-way ANOVA followed by LSD post-hoc test was used to assess the statistical significance of differences between groups. A value of *P *< 0.05 was considered to be statistically significant.

## Abbreviations

HHV-6: Human herpesvirus 6; CNS: Central nervous system; MS: Multiple sclerosis; AIDS: Acquired immunodeficiency syndrome; PHFA: Primary human fetal astrocyte; Bax: Bcl-2 associated X protein; Bcl-2: B cell lymphoma 2; AIF: Apoptosis-inducing factor; APAF1: Apoptotic protease activating factor 1; IAP: Inhibitor of apoptosis protein; IκBα: Nuclear factor of kappa light polypeptide gene enhancer in B-cells inhibitor, alpha; NF-κB: Nuclear factor kappa B; CPE: Cytopathic effect; hpi: Hours post-infection; PARP: Poly (ADP-ribose) polymerase; TNF- α: Tumor necrosisfactor-alpha; PI: Propidium iodide; PBS: Hosphate-buffered saline.

## Competing interests

The authors declare that they have no competing interests.

## Authors' contributions

BG, KY and WXH designed and directed studies, and were involved in the interpretation of the data and writing of manuscript. BG, GFZ and FZ carried out the cells culture and HHV-6 infection. BG, JFW and LYL participated in immunofluorescence assay and the western blot analysis. BG, DJF and CZ performed the flow-cytometry experiments. BG, JC and DDG performed the analysis of Caspase-8 and -9 activities and electron microscopy. CLD and HZ helped to modifiy the manuscript. All authors read and approved the final manuscript.
